# Supplementary material for: UPLC-G2Si-HDMS Untargeted Metabolomics for Identification of Yunnan Baiyao’s Metabolic Target in Promoting Blood Circulation and Removing Blood Stasis
Source: Molecules. 2022 May 17;27(10):3208. doi: 10.3390/molecules27103208 (PMC9143197; doi:10.3390/molecules27103208)
Supplement: Supplementary file 1 [file molecules-27-03208-s001.zip › molecules-1697256 proofed - supplementary.pdf]

UPLC-G2Si-HDMS Untargeted Metabolomics for Identification of Yunnan Baiyao's Metabolic Target in Promoting Blood Circulation and Removing Blood Stasis

Table S1. Secondary ion fragmentation information matching results.

1. Sumiki's acid:

Input:

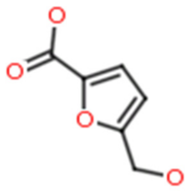	**ID (job)**	4
	**Mass (Da)**	142.0266
	**Formula**	$C_6H_6O_4$
	**DBE**	4

Experiment:

**Product ion(s) (Da)**	123.0335 105.9658 80.9571<br>+/- 0.1 in positive mode, structure filter off
**DBE**	0 to 50
**Electron count**	both
**Maximum H deficit**	6
**Fragment number of bonds**	4
**Scoring**	aromatic: 6, multiple: 4, ring: 2, phenyl: 8, other: 1<br>H-deficit: 0, hetero modifier: 0.5, max score: 16
**Order:**	mass
**Plot:**	show ◉ hide ○

Results:

**123.0335** ¬+ (-2H)	**105.9658** ¬+ (-2H)	**80.9571** ¬+ (+1H)

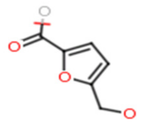 123.0082 (+25.3.mDa) (S:0.5, B:1) $C_6H_3O_3$ (-H_4O)	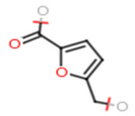 106.0055 (-39.7.mDa) (S:1.0, B:2) $C_6H_2O_2$ (-H_5O_2)	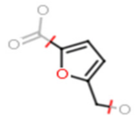 81.0340 (-76.9.mDa) (S:10.5, B:2) C_5H_5O (-CH_2O_3)

2. Imidaz-ole-4-acetaldehyde:

Input:

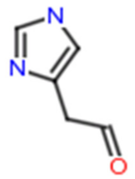	**ID (job)**	10
	**Mass (Da)**	110.0480
	**Formula**	$C_5H_6N_2O$
	**DBE**	4

Experiment:

**Product ion(s) (Da)**	93.0945 79.9555<br>+/- 0.1 in positive mode, structure filter off
**DBE**	0 to 50
**Electron count**	both
**Maximum H deficit**	6
**Fragment number of bonds**	4
**Scoring**	aromatic: 6, multiple: 4, ring: 2, phenyl: 8, other: 1<br>H-deficit: 0, hetero modifier: 0.5, max score: 16
**Order:**	mass
**Plot:**	show ◉ hide ○

Results:

**93.0945**	¬+ (-1H)	**79.9555**	¬+ (-1H)

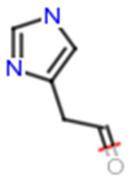<br>93.0453 (+49.2.mDa) (S:2.0, B:1)<br>$C_5H_5N_2$ (-H_2O)	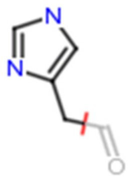<br>80.0374 (-81.9.mDa) (S:1.0, B:1)<br>$C_4H_4N_2$ (-CH_3O)

3. Gam-ma-delta-Dioxoval eric acid:

Input:

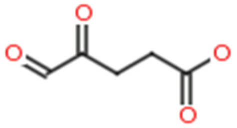	**ID (job)**	65
	**Mass (Da)**	130.0266
	**Formula**	$C_5H_6O_4$
	**DBE**	3

Experiment:

**Product ion(s) (Da)**	115.9131 85.0256 99.9185<br>+/- 0.1 in positive mode, structure filter off
**DBE**	0 to 50
**Electron count**	both
**Maximum H deficit**	6
**Fragment number of bonds**	4
**Scoring**	aromatic: 6, multiple: 4, ring: 2, phenyl: 8, other: 1<br>H-deficit: 0, hetero modifier: 0.5, max score: 16
**Order:**	mass
**Plot:**	show ⦿ hide ○

Results:

**99.9185**	¬+ (-1H)	**85.0256**	¬+ (+1H)

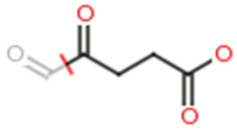<br>100.0160 (-97.5.mDa) (S:1.0, B:1)<br>$C_4H_4O_3$ (-CH_3O)	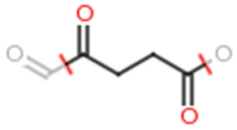<br>85.0290 (-3.4.mDa) (S:1.5, B:2)<br>$C_4H_5O_2$ (-CH_2O_2)

4. Glutaconic acid:

Input:

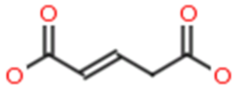	**ID (job)**	40
	**Mass (Da)**	130.0266
	**Formula**	$C_5H_6O_4$
	**DBE**	3

Experiment:

**Product ion(s) (Da)**	111.0621 83.0652<br>+/- 0.1 in positive mode, structure filter off
**DBE**	0 to 50
**Electron count**	both
**Maximum H deficit**	6
**Fragment number of bonds**	4
**Scoring**	aromatic: 6, multiple: 4, ring: 2, phenyl: 8, other: 1<br>H-deficit: 0, hetero modifier: 0.5, max score: 16
**Order:**	mass
**Plot:**	show ◻ hide ◻

Results:

**111.0621** ¬+ (-2H)<br>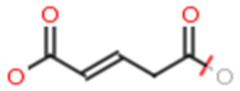<br>111.0082 (+53.9.mDa) (S:0.5, B:1)<br>$C_5H_3O_3$ (-H_4O)	**83.0652** ¬+ (-2H)<br>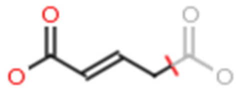<br>83.0133 (+51.9.mDa) (S:1.0, B:1)<br>$C_4H_3O_2$ (-CH_4O_2)

5. Oxoadipic acid:

Input:

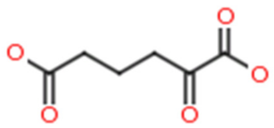	**ID (job)**	86
	**Mass (Da)**	160.0372
	**Formula**	$C_6H_8O_5$
	**DBE**	3

Experiment:

**Product ion(s) (Da)**	115.0353 121.9970 136.0336 98.0222<br>+/- 0.1 in positive mode, structure filter off
**DBE**	0 to 50
**Electron count**	both
**Maximum H deficit**	6
**Fragment number of bonds**	4
**Scoring**	aromatic: 6, multiple: 4, ring: 2, phenyl: 8, other: 1<br>H-deficit: 0, hetero modifier: 0.5, max score: 16
**Order:**	mass
**Plot:**	show hide

Results:

**115.0353** ¬+ (+0H)	**98.0222** ¬+ (+0H)
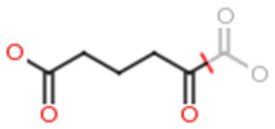	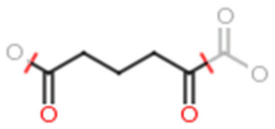
115.0395 (-4.2.mDa) (S:1.0, B:1)<br>$C_5H_7O_3$ ($-CH_2O_2$)	98.0368 (-14.6.mDa) (S:1.5, B:2)<br>$C_5H_6O_2$ ($-CH_3O_3$)

6. L-2-Hydroxyglutaric acid:

Input:

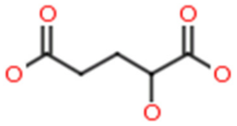

**ID (job)**	78
**Mass (Da)**	148.0372
**Formula**	$C_5H_8O_5$
**DBE**	2

Experiment:

**Product ion(s) (Da)**	103.0378 123.0407 85.0239 87.0050<br>+/- 0.1 in positive mode, structure filter off
**DBE**	0 to 50
**Electron count**	both
**Maximum H deficit**	6
**Fragment number of bonds**	4
**Scoring**	aromatic: 6, multiple: 4, ring: 2, phenyl: 8, other: 1<br>H-deficit: 0, hetero modifier: 0.5, max score: 16
**Order:**	mass
**Plot:**	show hide

Results:

**103.0378** ¬+ (+0H)<br>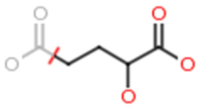<br>103.0395 (-1.7.mDa) (S:1.0, B:1) $C_4H_7O_3$ (-CH_2O_2)	**87.0050** ¬+ (+1H)<br>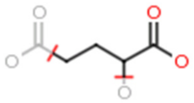<br>87.0446 (-39.6.mDa) (S:1.5, B:2) $C_4H_7O_2$ (-CH_2O_3)	**85.0239** ¬+ (-1H)<br>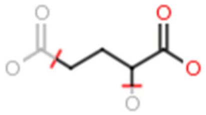<br>85.0290 (-5.1.mDa) (S:1.5, B:2) $C_4H_5O_2$ (-CH_4O_3)

7. DHAP (8:0):

Input:

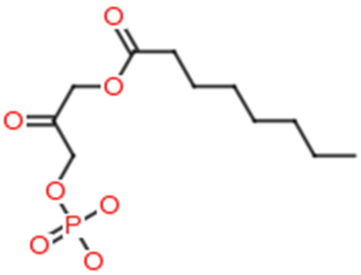	**ID (job)**	6
	**Mass (Da)**	296.1025
	**Formula**	$C_{11}H_{21}O_7P$
	**DBE**	3

Experiment:

**Product ion(s) (Da)**	159.0454 175.0756 253.0503 295.1271<br>+/- 0.1 in negative mode, structure filter off
**DBE**	0 to 50
**Electron count**	both
**Maximum H deficit**	6
**Fragment number of bonds**	4
**Scoring**	aromatic: 6, multiple: 4, ring: 2, phenyl: 8, other: 1<br>H-deficit: 0, hetero modifier: 0.5, max score: 16
**Order:**	mass
**Plot:**	show hide

Results:

**253.0503** ¬- (+2H)	**175.0756** ¬- (+5H)	**159.0454** ¬- (+3H)
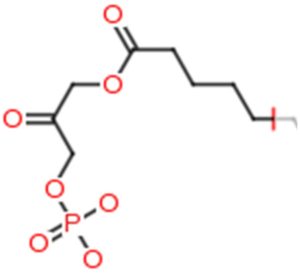	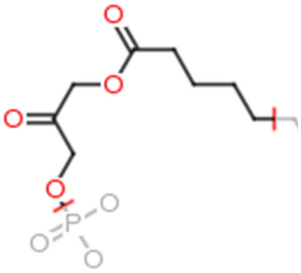	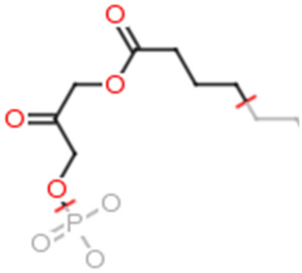
253.0477 (+2.6.mDa)<br>(S:1.0, B:1)<br>$C_8H_{14}O_7P$ (-C_3H_6)	175.0970 (-21.4.mDa)<br>(S:2.0, B:2)<br>$C_8H_{15}O_4$ (-$C_3H_5O_3P$)	159.0657 (-20.3.mDa)<br>(S:2.0, B:2)<br>$C_7H_{11}O_4$ (-$C_4H_9O_3P$)

8. Vinylacetylglycine:

Input:

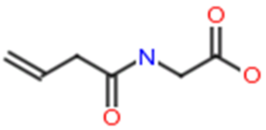	**ID (job)**	37
	**Mass (Da)**	143.0582
	**Formula**	$C_6H_9NO_3$
	**DBE**	3

Experiment:

**Product ion(s) (Da)**	124.0346 143.0363 83.0675 98.0528<br>+/- 0.1 in positive mode, structure filter off
**DBE**	0 to 50
**Electron count**	both
**Maximum H deficit**	6
**Fragment number of bonds**	4
**Scoring**	aromatic: 6, multiple: 4, ring: 2, phenyl: 8, other: 1<br>H-deficit: 0, hetero modifier: 0.5, max score: 16
**Order:**	mass
**Plot:**	show ☐ hide ☐

Results:

**124.0346** ¬+ (-2H)	**98.0528** ¬+ (+0H)	**83.0675** ¬+ (-1H)

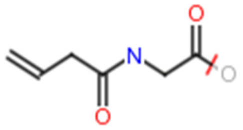 124.0399 (-5.3.mDa) (S:0.5, B:1) $C_6H_6NO_2$ (-H_4O)	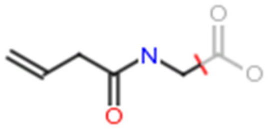 98.0606 (-7.8.mDa) (S:1.0, B:1) C_5H_8NO (-CH_2O_2)	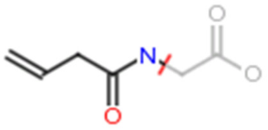 83.0371 (+30.4.mDa) (S:0.5, B:1) C_4H_5NO (-$C_2H_5O_2$)

9. Allysine:

Input:

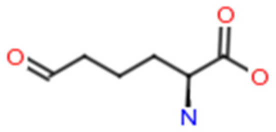	**ID (job)**	55
	**Mass (Da)**	145.0739
	**Formula**	$C_6H_{11}NO_3$
	**DBE**	2

Experiment:

**Product ion(s) (Da)**	85.0265 87.0020<br>+/- 0.1 in positive mode, structure filter off
**DBE**	0 to 50
**Electron count**	both
**Maximum H deficit**	6
**Fragment number of bonds**	4
**Scoring**	aromatic: 6, multiple: 4, ring: 2, phenyl: 8, other: 1<br>H-deficit: 0, hetero modifier: 0.5, max score: 16
**Order:**	mass
**Plot:**	show ◉ hide ○

Results:

**87.0020**	¬+ (+1H)	**85.0265**	¬+ (-1H)

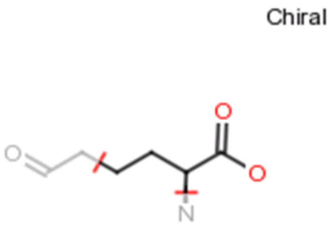 87.0446 (-42.6.mDa) (S:1.5, B:2) $C_4H_7O_2$ (-C_2H_5NO)	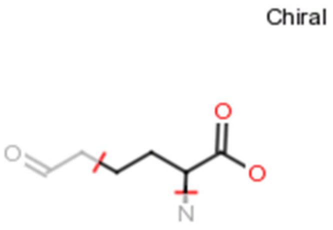 85.0290 (-2.5.mDa) (S:1.5, B:2) $C_4H_5O_2$ (-C_2H_7NO)

10. Thymidine:

Input:

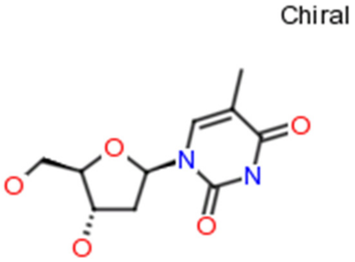	**ID (job)**	71
	**Mass (Da)**	242.0903
	**Formula**	$C_{10}H_{14}N_2O_5$
	**DBE**	6

Experiment:

**Product ion(s) (Da)**	108.0213 121.0293 131.0824 203.0009 242.0780<br>+/- 0.1 in negative mode, structure filter off
**DBE**	0 to 50
**Electron count**	both
**Maximum H deficit**	6
**Fragment number of bonds**	4
**Scoring**	aromatic: 6, multiple: 4, ring: 2, phenyl: 8, other: 1<br>H-deficit: 0, hetero modifier: 0.5, max score: 16
**Order:**	mass
**Plot:**	show hide

Results:

**203.000 9** ¬- (+3H) 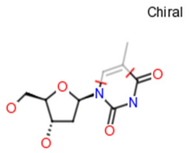 203.0668 (-65.9.mDa) (S:3.0, B:2) $C_7H_{11}N_2O_5$ (-C_3H_2)	**131.082 4** ¬- (+5H) 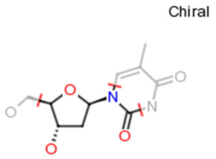 131.0582 (+24.2.mDa) (S:3.0, B:3) $C_5H_9NO_3$ (-$C_5H_4NO_2$)	**108.021 3** ¬- (-1H) 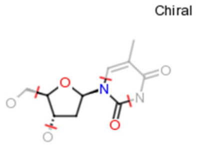 108.0086 (+12.7.mDa) (S:3.5, B:4) $C_5H_2NO_2$ (-$C_5H_{11}NO_3$)

11. Gentisate aldehyde:

Input:

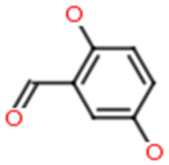	**ID (job)**	11
	**Mass (Da)**	138.0317
	**Formula**	$C_7H_6O_3$
	**DBE**	5

Experiment:

**Product ion(s) (Da)**	121.0161 109.0313<br>+/- 0.1 in positive mode, structure filter off
**DBE**	0 to 50
**Electron count**	both
**Maximum H deficit**	6
**Fragment number of bonds**	4
**Scoring**	aromatic: 6, multiple: 4, ring: 2, phenyl: 8, other: 1<br>H-deficit: 0, hetero modifier: 0.5, max score: 16
**Order:**	mass
**Plot:**	show ◉ hide ○

Results:

**121.0161** ¬+ (-1H)	**109.0313** ¬+ (+0H)
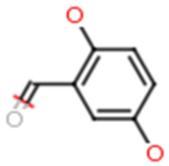	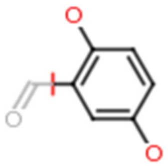
121.0290 (-12.9.mDa) (S:2.0, B:1)<br>$C_7H_5O_2$ (-H_2O)	109.0290 (+2.3.mDa) (S:10.0, B:1)<br>$C_6H_5O_2$ (-CH_2O)

12. 2-Octenedioic acid:

Input:

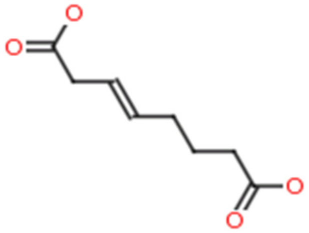	**ID (job)**	96
	**Mass (Da)**	172.0736
	**Formula**	$C_8H_{12}O_4$
	**DBE**	4

Experiment:

**Product ion(s) (Da)**	111.0816 129.0916 171.0653 86.9758<br>+/- 0.1 in negative mode, structure filter off
**DBE**	0 to 50
**Electron count**	both
**Maximum H deficit**	6
**Fragment number of bonds**	4
**Scoring**	aromatic: 6, multiple: 4, ring: 2, phenyl: 8, other: 1<br>H-deficit: 0, hetero modifier: 0.5, max score: 16
**Order:**	mass
**Plot:**	show ◉ hide ○

Results:

**129.0916** ¬- (+4H)	**111.0816** ¬- (+0H)	**86.9758** ¬- (+4H)
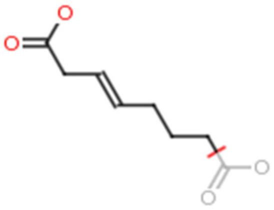	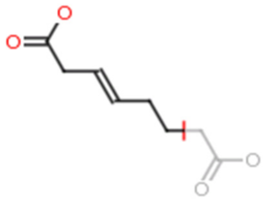	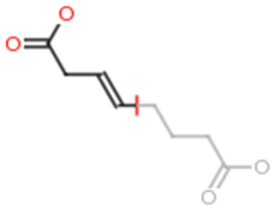
129.0916 (+0.0.mDa) (S:1.0, B:1) $C_7H_{13}O_2$ (-CO_2)	111.0446 (+37.0.mDa) (S:1.0, B:1) $C_6H_7O_2$ (-$C_2H_4O_2$)	87.0446 (-68.8.mDa) (S:1.0, B:1) $C_4H_7O_2$ (-$C_4H_4O_2$)

13. Enkephalin L:

Input:

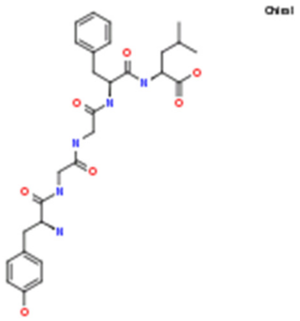	**ID (job)**	85
	**Mass (Da)**	555.2693
	**Formula**	$C_{28}H_{37}N_5O_7$
	**DBE**	14

Experiment:

**Product ion(s) (Da)**	130.0867 169.0723 236.1032 554.2607 +/- 0.1 in negative mode, structure filter off
**DBE**	0 to 50
**Electron count**	both
**Maximum H deficit**	6
**Fragment number of bonds**	4
**Scoring**	aromatic: 6, multiple: 4, ring: 2, phenyl: 8, other: 1 H-deficit: 0, hetero modifier: 0.5, max score: 16
**Order:**	mass
**Plot:**	show ◉ hide ○

Results:

**236.1032** ¬- (-2H)	**169.0723** ¬- (+1H)	**130.0867** ¬- (+4H)

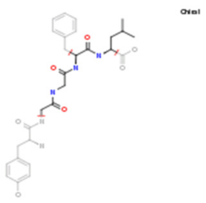 236.1035 (-0.3.mDa) (S:2.5, B:3) $C_{11}H_{14}N_3O_3$ (-$C_{17}H_{22}N_2O_4$)	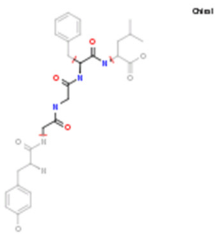 169.0487 (+23.6.mDa) (S:2.0, B:3) $C_6H_7N_3O_3$ (-$C_{22}H_{29}N_2O_4$)	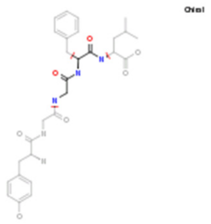 130.0617 (+25.0.mDa) (S:2.0, B:3) $C_4H_8N_3O_2$ (-$C_{24}H_{28}N_2O_5$)

14. Normetanephrine:

Input:

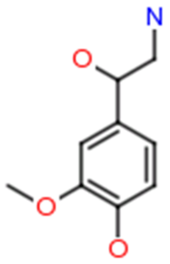	**ID (job)**	44
	**Mass (Da)**	183.0895
	**Formula**	$C_9H_{13}NO_3$
	**DBE**	4

Experiment:

**Product ion(s) (Da)**	166.1053 150.1259 137.9975 131.0403<br>+/- 0.1 in positive mode, structure filter off
**DBE**	0 to 50
**Electron count**	both
**Maximum H deficit**	6
**Fragment number of bonds**	4
**Scoring**	aromatic: 6, multiple: 4, ring: 2, phenyl: 8, other: 1<br>H-deficit: 0, hetero modifier: 0.5, max score: 16
**Order:**	mass
**Plot:**	show ☐ hide ☐

Results:

**166.1053** ¬+ (-1H)<br>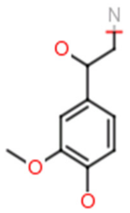<br>166.0630 (+42.3.mDa) (S:0.5, B:1)<br>$C_9H_{10}O_3$ (-H_4N)	**150.1259** ¬+ (+0H)<br>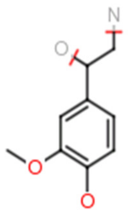<br>150.0681 (+57.8.mDa) (S:1.0, B:2)<br>$C_9H_{10}O_2$ (-H_4NO)	**137.9975** ¬+ (+3H)<br>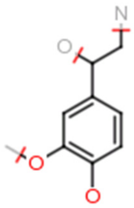<br>138.0681 (-70.6.mDa) (S:1.5, B:3)<br>$C_8H_{10}O_2$ (-CH_4NO)	**131.0403** ¬+ (-4H)<br>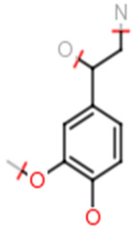<br>131.0133 (+27.0.mDa) (S:1.5, B:3)<br>$C_8H_3O_2$ (-$CH_{11}NO$)

15. 2-Methyl-3-ketovaleric acid:

Input:

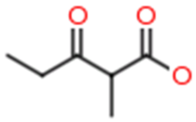	**ID (job)**	42
	**Mass (Da)**	130.0630
	**Formula**	$C_6H_{10}O_3$
	**DBE**	2

Experiment:

**Product ion(s) (Da)**	96.9583 79.9693<br>+/- 0.1 in positive mode, structure filter off
**DBE**	0 to 50
**Electron count**	both
**Maximum H deficit**	6
**Fragment number of bonds**	4
**Scoring**	aromatic: 6, multiple: 4, ring: 2, phenyl: 8, other: 1<br>H-deficit: 0, hetero modifier: 0.5, max score: 16
**Order:**	mass
**Plot:**	show ◯ hide ◯

Results:

**96.9583** ¬+ (-1H)<br>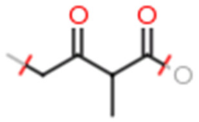<br>97.0290 (-70.7.mDa) (S:1.5, B:2)<br>$C_5H_5O_2$ (-CH_6O)	**79.9693** ¬·+ (-3H)<br>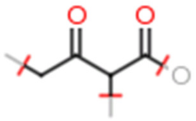<br>79.9898 (-20.5.mDa) (S:2.5, B:3)<br>C_4O_2 (-$C_2H_{11}O$)

16. Enol-phenylpyruvate:

Input:

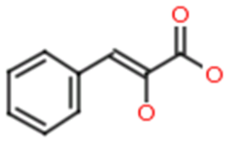	**ID (job)**	38
	**Mass (Da)**	164.0473
	**Formula**	$C_9H_8O_3$
	**DBE**	6

Experiment:

**Product ion(s) (Da)**	164.9961 147.0202 119.0432 92.1066<br>+/- 0.1 in positive mode, structure filter off
**DBE**	0 to 50
**Electron count**	both
**Maximum H deficit**	6
**Fragment number of bonds**	4
**Scoring**	aromatic: 6, multiple: 4, ring: 2, phenyl: 8, other: 1<br>H-deficit: 0, hetero modifier: 0.5, max score: 16
**Order:**	mass
**Plot:**	show ◯ hide ◯

Results:

**147.0202** ¬+ (+0H)	**119.0432** ¬+ (+0H)	**92.1066** ¬+ (+2H)
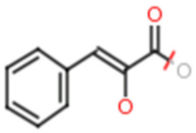	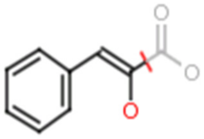	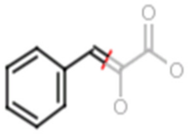
147.0446 (-24.4.mDa) (S:0.5, B:1)<br>$C_9H_7O_2$ (-H_2O)	119.0497 (-6.5.mDa) (S:1.0, B:1)<br>C_8H_7O (-CH_2O_2)	92.0626 (+44.0.mDa) (S:4.0, B:1)<br>C_7H_8 (-C_2HO_3)

17. (4-hydroxyphenyl)acetaldehyde:

Input:

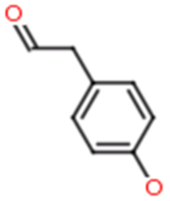	**ID (job)**	41
	**Mass (Da)**	136.0524
	**Formula**	$C_8H_8O_2$
	**DBE**	5

Experiment:

**Product ion(s) (Da)**	136.0288 108.0139 93.0328<br>+/- 0.1 in positive mode, structure filter off
**DBE**	0 to 50
**Electron count**	both
**Maximum H deficit**	6
**Fragment number of bonds**	4
**Scoring**	aromatic: 6, multiple: 4, ring: 2, phenyl: 8, other: 1<br>H-deficit: 0, hetero modifier: 0.5, max score: 16
**Order:**	mass
**Plot:**	show ▢ hide ▢

Results:

**136.0288** ¬+ (+0H)<br>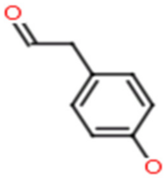<br>136.0524 (-23.6.mDa) (S:8.0, B:0)<br>$C_8H_8O_2$ (-H)	**108.0139** ¬+ (+1H)<br>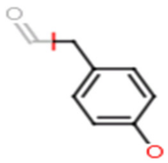<br>108.0575 (-43.6.mDa) (S:1.0, B:1)<br>C_7H_8O (-CHO)	**93.0328** ¬+ (+0H)<br>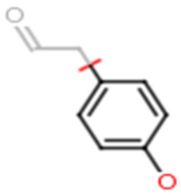<br>93.0340 (-1.2.mDa) (S:10.0, B:1)<br>C_6H_5O (-C_2H_4O)

18. Pimelic acid:

Input:

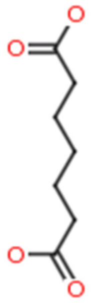	**ID (job)**	12
	**Mass (Da)**	160.0736
	**Formula**	$C_7H_{12}O_4$
	**DBE**	2

Experiment:

**Product ion(s) (Da)**	144.0050 125.0203 96.0089<br>+/- 0.1 in positive mode, structure filter off
**DBE**	0 to 50
**Electron count**	both
**Maximum H deficit**	6
**Fragment number of bonds**	4
**Scoring**	aromatic: 6, multiple: 4, ring: 2, phenyl: 8, other: 1<br>H-deficit: 0, hetero modifier: 0.5, max score: 16
**Order:**	mass
**Plot:**	show ◉ hide ○

Results:

**144.0050** ¬+ (+1H)<br>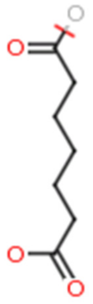<br>144.0786 (-73.6.mDa)<br>(S:0.5, B:1)<br>$C_7H_{12}O_3$ (-HO)	**125.0203** ¬+ (-1H)<br>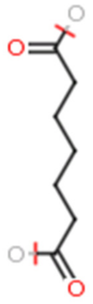<br>125.0603 (-40.0.mDa)<br>(S:1.0, B:2)<br>$C_7H_9O_2$ (-H_4O_2)	**96.0089** ¬+ (-2H)<br>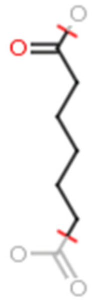<br>96.0575 (-48.6.mDa)<br>(S:1.5, B:2)<br>C_6H_8O (-CH_5O_3)

19. (+) - (1R,2R)-1,2-Diphenylethane-1,2-diol:

Input:

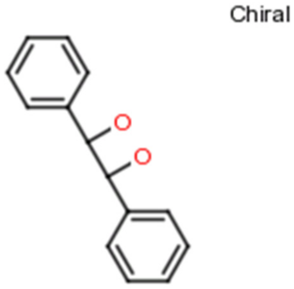	**ID (job)**	7
	**Mass (Da)**	214.0994
	**Formula**	$C_{14}H_{14}O_2$
	**DBE**	8

Experiment:

**Product ion(s) (Da)**	135.0413 137.0956 181.0764<br>+/- 0.1 in positive mode, structure filter off
**DBE**	0 to 50
**Electron count**	both
**Maximum H deficit**	6
**Fragment number of bonds**	4
**Scoring**	aromatic: 6, multiple: 4, ring: 2, phenyl: 8, other: 1<br>H-deficit: 0, hetero modifier: 0.5, max score: 16
**Order:**	mass
**Plot:**	show ◉ hide ○

Results:

**137.0956** ¬+ (+0H)	**135.0413** ¬+ (-2H)

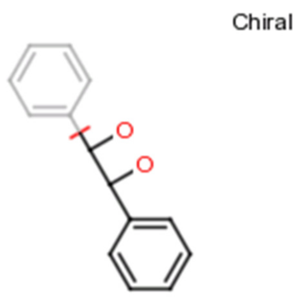<br>137.0603 (+35.3.mDa) (S:10.0, B:1)<br>$C_8H_9O_2$ ($-C_6H_6$)	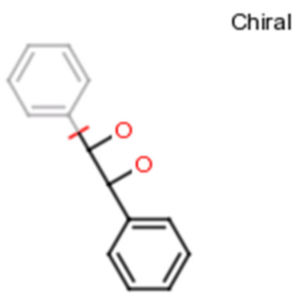<br>135.0446 (-3.3.mDa) (S:10.0, B:1)<br>$C_8H_7O_2$ ($-C_6H_8$)

20. 5-L-Glutamyl-taurine:

Input:

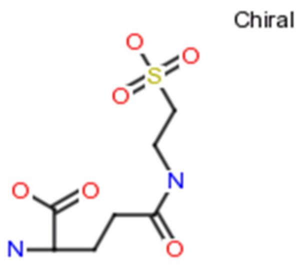

**ID (job)**	12
**Mass (Da)**	254.0573
**Formula**	$C_7H_{14}N_2O_6S$
**DBE**	3

Experiment:

**Product ion(s) (Da)**	143.0500 165.1283 209.0609 253.0504<br>+/- 0.1 in negative mode, structure filter off
**DBE**	0 to 50
**Electron count**	both
**Maximum H deficit**	6
**Fragment number of bonds**	4
**Scoring**	aromatic: 6, multiple: 4, ring: 2, phenyl: 8, other: 1<br>H-deficit: 0, hetero modifier: 0.5, max score: 16
**Order:**	mass
**Plot:**	show ◉ hide ○

Results:

**209.0609**	¬- (+2H)	**165.1283**	¬- (+4H)	**143.0500**	¬- (-2H)

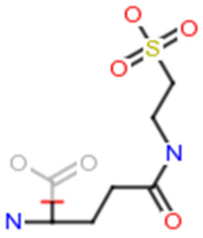<br>209.0596 (+1.3.mDa)<br>(S:1.0, B:1)<br>$C_6H_{13}N_2O_4S$ (-CO_2)	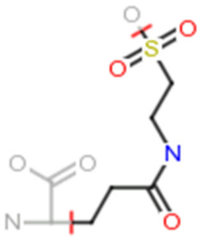<br>165.0460 (+82.3.mDa)<br>(S:2.0, B:2)<br>$C_5H_{11}NO_3S$ (-$C_2H_2NO_3$)	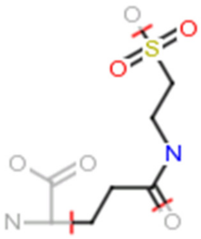<br>143.0041 (+45.9.mDa)<br>(S:4.0, B:3)<br>$C_5H_5NO_2S$ (-$C_2H_8NO_4$)

21. Thymi-dine5'-triphosphate:

Input:

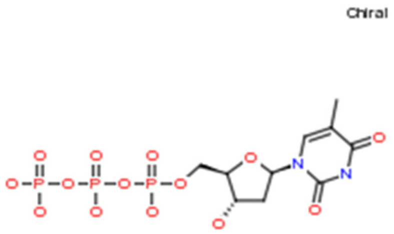	**ID (job)**	76
	**Mass (Da)**	481.9893
	**Formula**	$C_{10}H_{17}N_2O_{14}P_3$
	**DBE**	6

Experiment:

**Product ion(s) (Da)**	162.0537 212.0024 242.0126 480.9731<br>+/- 0.1 in negative mode, structure filter off
**DBE**	0 to 50
**Electron count**	both
**Maximum H deficit**	6
**Fragment number of bonds**	4
**Scoring**	aromatic: 6, multiple: 4, ring: 2, phenyl: 8, other: 1<br>H-deficit: 0, hetero modifier: 0.5, max score: 16
**Order:**	mass
**Plot:**	show ◉ hide ○

Results:

**242.0126** ¬- (+3H)	**212.0024** ¬- (+4H)	**162.0537** ¬- (-2H)

<table>
<tr>
<td>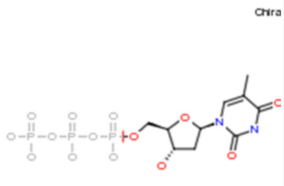

242.0903 (-77.7.mDa)
(S:1.0, B:1)
$C_{10}H_{14}N_2O_5$ ($-H_2O_9P_3$)</td>
<td>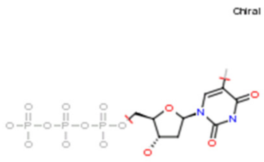

212.0797 (-77.3.mDa)
(S:1.5, B:2)
$C_9H_{12}N_2O_4$ ($-CH_4O_{10}P_3$)</td>
<td>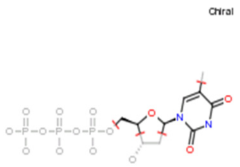

162.0065 (+47.2.mDa)
(S:5.5, B:4)
$C_7H_2N_2O_3$ ($-C_3H_{14}O_{11}P_3$)</td>
</tr>
</table>

22. D-Leucine:

Input:

<table>
<tr><td rowspan="4">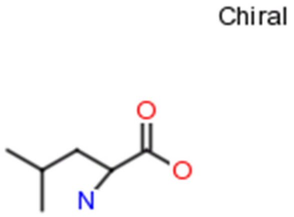
</td><td>ID (job)</td><td>13</td></tr>
<tr><td>Mass (Da)</td><td>131.0946</td></tr>
<tr><td>Formula</td><td>$C_6H_{13}NO_2$</td></tr>
<tr><td>DBE</td><td>1</td></tr>
</table>

Experiment:

**Product ion(s) (Da)**	89.0084 72.9893<br>+/- 0.1 in positive mode, structure filter off
**DBE**	0 to 50
**Electron count**	both
**Maximum H deficit**	6
**Fragment number of bonds**	4
**Scoring**	aromatic: 6, multiple: 4, ring: 2, phenyl: 8, other: 1<br>H-deficit: 0, hetero modifier: 0.5, max score: 16
**Order:**	mass
**Plot:**	show ◉ hide ○

Results:

**89.0084** ¬+ (+1H)<br>Chiral<br>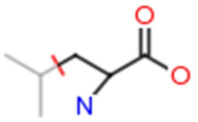<br>89.0477 (-39.3.mDa) (S:1.0, B:1)<br>$C_3H_7NO_2$ (-C_3H_7)	**72.9893** ¬+ (+2H)<br>Chiral<br>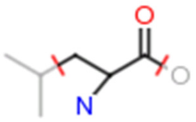<br>73.0528 (-63.5.mDa) (S:1.5, B:2)<br>C_3H_7NO (-C_3H_7O)

23. 3-Oxodecanoic acid:

Input:

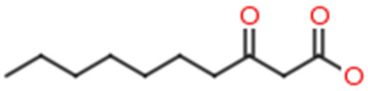	**ID (job)**	75
	**Mass (Da)**	186.1256
	**Formula**	$C_{10}H_{18}O_3$
	**DBE**	3

Experiment:

**Product ion(s) (Da)**	109.0276 125.1014 141.0911 185.1135 79.9580<br>+/- 0.1 in negative mode, structure filter off
**DBE**	0 to 50
**Electron count**	both
**Maximum H deficit**	6
**Fragment number of bonds**	4
**Scoring**	aromatic: 6, multiple: 4, ring: 2, phenyl: 8, other: 1<br>H-deficit: 0, hetero modifier: 0.5, max score: 16
**Order:**	mass
**Plot:**	show ◉ hide ○

Results:

**141.0911** ¬- (+0H)<br>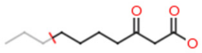<br>141.0552 (+35.9.mDa)<br>(S:1.0, B:1)<br>$C_7H_9O_3$ (-C_3H_8)	**125.1014** ¬- (+1H)<br>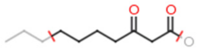<br>125.0603 (+41.1.mDa)<br>(S:1.5, B:2)<br>$C_7H_9O_2$ (-C_3H_8O)	**109.0276** ¬- (-1H)<br>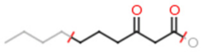<br>109.0290 (-1.4.mDa)<br>(S:1.5, B:2)<br>$C_6H_5O_2$ (-$C_4H_{12}O$)	**79.9580** ¬- (+0H)<br>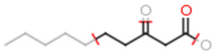<br>80.0262 (-68.2.mDa)<br>(S:3.5, B:3)<br>C_5H_4O (-$C_5H_{13}O_2$)

24. 3-Hydroxycapric acid:

Input:

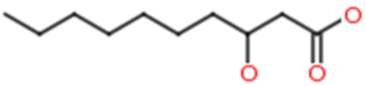	**ID (job)**	43
	**Mass (Da)**	188.1412
	**Formula**	$C_{10}H_{20}O_3$
	**DBE**	1

Experiment:

**Product ion(s) (Da)**	151.0831 145.0560 135.0472 124.0039<br>+/- 0.1 in positive mode, structure filter off
**DBE**	0 to 50
**Electron count**	both
**Maximum H deficit**	6
**Fragment number of bonds**	4
**Scoring**	aromatic: 6, multiple: 4, ring: 2, phenyl: 8, other: 1<br>H-deficit: 0, hetero modifier: 0.5, max score: 16
**Order:**	mass
**Plot:**	show ▢ hide ▢

Results:

**151.0831** ¬+ (-3H) 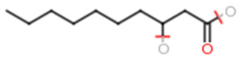 151.1123 (-29.2.mDa) (S:1.0, B:2) $C_{10}H_{15}O$ (-H_6O_2)	**145.0560** ¬+ (+3H) 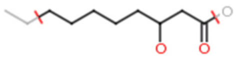 145.1229 (-66.9.mDa) (S:1.5, B:2) $C_8H_{17}O_2$ (-C_2H_4O)	**124.0039** ¬+ (-1H) 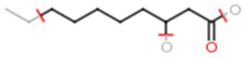 124.0888 (-84.9.mDa) (S:2.0, B:3) $C_8H_{12}O$ (-$C_2H_9O_2$)

25. Prostaglandin E2:

Input:

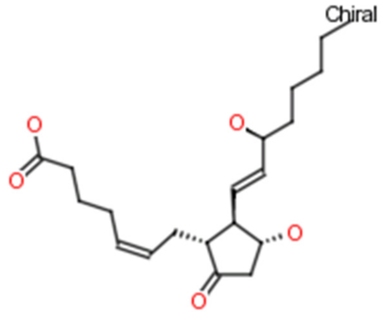	**ID (job)**	62
	**Mass (Da)**	352.2250
	**Formula**	$C_{20}H_{32}O_5$
	**DBE**	6

Experiment:

**Product ion(s) (Da)**	115.0025 162.0184 241.9754 351.2107<br>+/- 0.1 in negative mode, structure filter off
**DBE**	0 to 50
**Electron count**	both
**Maximum H deficit**	6
**Fragment number of bonds**	4
**Scoring**	aromatic: 6, multiple: 4, ring: 2, phenyl: 8, other: 1<br>H-deficit: 0, hetero modifier: 0.5, max score: 16
**Order:**	mass
**Plot:**	show hide

Results:

**351.2107** ¬- (+1H)	**162.0184** ¬- (+0H)	**115.0025** ¬- (+5H)
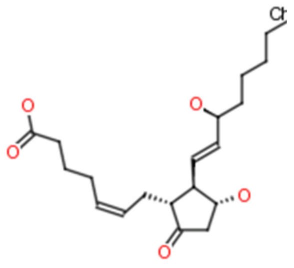	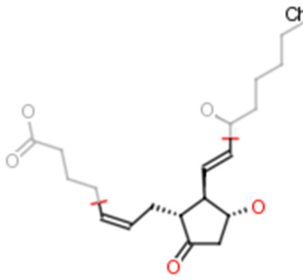	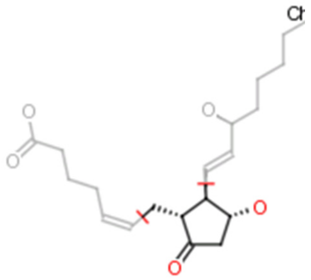
351.2171 (-6.4.mDa)<br>(S:2.0, B:0)<br>$C_{20}H_{31}O_5$ (-none)	162.0681 (-49.7.mDa)<br>(S:2.0, B:2)<br>$C_{10}H_{10}O_2$ (-$C_{10}H_{21}O_3$)	115.0759 (-73.4.mDa)<br>(S:2.0, B:2)<br>$C_6H_{11}O_2$ (-$C_{14}H_{20}O_3$)

26. (+)-trans-Carveol:

Input:

Chiral	**ID (job)**	39
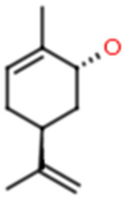	**Mass (Da)**	152.1201
	**Formula**	$C_{10}H_{16}O$
	**DBE**	3

Experiment:

**Product ion(s) (Da)**	136.0390 122.0321 94.0446<br>+/- 0.1 in positive mode, structure filter off
**DBE**	0 to 50
**Electron count**	both
**Maximum H deficit**	6
**Fragment number of bonds**	4
**Scoring**	aromatic: 6, multiple: 4, ring: 2, phenyl: 8, other: 1<br>H-deficit: 0, hetero modifier: 0.5, max score: 16
**Order:**	mass
**Plot:**	show hide

Results:

**136.0390** ¬+ (+1H)<br>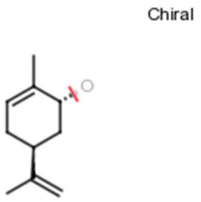<br><br>136.1252 (-86.2.mDa) (S:0.5, B:1)<br>$C_{10}H_{16}$ (-HO)	**122.0321** ¬·+ (+2H)<br>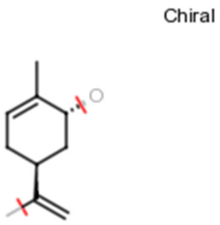<br><br>122.1096 (-77.4.mDa) (S:1.5, B:2)<br>C_9H_{14} (-CH_3O)	**94.0446** ¬+ (+0H)<br>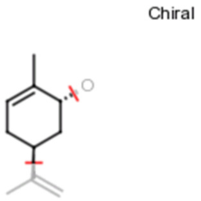<br><br>94.0783 (-33.6.mDa) (S:1.5, B:2)<br>C_7H_{10} (-C_3H_7O)

27. trans-Dec-2-enoic acid:

Input:

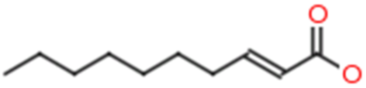	**ID (job)**	35
	**Mass (Da)**	170.1307
	**Formula**	$C_{10}H_{18}O_2$
	**DBE**	2

Experiment:

**Product ion(s) (Da)**	140.0370 127.0250 97.0009 83.0496<br>+/- 0.1 in positive mode, structure filter off
**DBE**	0 to 50
**Electron count**	both
**Maximum H deficit**	6
**Fragment number of bonds**	4
**Scoring**	aromatic: 6, multiple: 4, ring: 2, phenyl: 8, other: 1<br>H-deficit: 0, hetero modifier: 0.5, max score: 16
**Order:**	mass
**Plot:**	show ◉ hide ○

Results:

**140.0370** ¬+ (-1H)<br>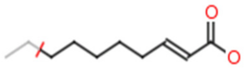<br>140.0837 (-46.7.mDa) (S:1.0, B:1)<br>$C_8H_{12}O_2$ (-C_2H_7)	**127.0250** ¬+ (+0H)<br>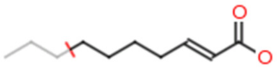<br>127.0759 (-50.9.mDa) (S:1.0, B:1)<br>$C_7H_{11}O_2$ (-C_3H_8)	**97.0009** ¬+ (-2H)<br>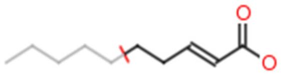<br>97.0290 (-28.1.mDa) (S:1.0, B:1)<br>$C_5H_5O_2$ (-C_5H_{14})	**83.0496** ¬+ (-2H)<br>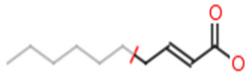<br>83.0133 (+36.3.mDa) (S:1.0, B:1)<br>$C_4H_3O_2$ (-C_6H_{16})

28. Acetylcysteine:

Input:

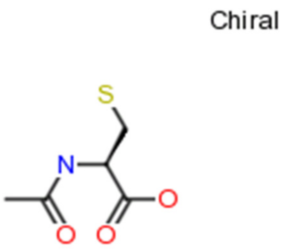	**ID (job)**	14
	**Mass (Da)**	163.0303
	**Formula**	$C_5H_9NO_3S$
	**DBE**	2

Experiment:

**Product ion(s) (Da)**	147.0233 133.0462 102.0408<br>+/- 0.1 in positive mode, structure filter off
**DBE**	0 to 50
**Electron count**	both
**Maximum H deficit**	6
**Fragment number of bonds**	4
**Scoring**	aromatic: 6, multiple: 4, ring: 2, phenyl: 8, other: 1<br>H-deficit: 0, hetero modifier: 0.5, max score: 16
**Order:**	mass
**Plot:**	show ◙ hide ◻

Results:

**147.0233** ¬+ (+1H)	**133.0462** ¬+ (+2H)	**102.0408** ¬+ (-1H)
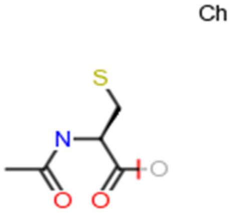	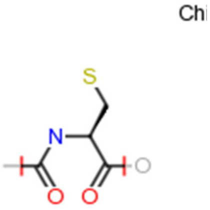	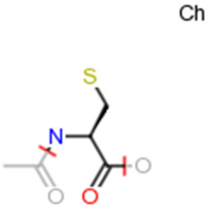
147.0354 (-12.1.mDa)	133.0198 (+26.4.mDa)	102.0014 (+39.4.mDa)
(S:0.5, B:1)	(S:1.5, B:2)	(S:1.0, B:2)
$C_5H_9NO_2S$ (-HO)	$C_4H_7NO_2S$ (-CH_3O)	C_3H_4NOS (-$C_2H_6O_2$)
